# Mini Review: Clinical Routine Microbiology in the Era of Automation and Digital Health

**DOI:** 10.3389/fcimb.2020.582028

**Published:** 2020-11-30

**Authors:** Stefano Leo, Abdessalam Cherkaoui, Gesuele Renzi, Jacques Schrenzel

**Affiliations:** ^1^ Genomic Research Laboratory, Division of Infectious Diseases, Department of Medicine, Geneva University Hospitals and University of Geneva, Geneva, Switzerland; ^2^ Bacteriology Laboratory, Division of Laboratory Medicine, Department of Diagnostics, Geneva University Hospitals, Geneva, Switzerland

**Keywords:** clinical microbiology, machine learning, laboratory automation, diagnostics, next-generation sequencing

## Abstract

Clinical microbiology laboratories are the first line to combat and handle infectious diseases and antibiotic resistance, including newly emerging ones. Although most clinical laboratories still rely on conventional methods, a cascade of technological changes, driven by digital imaging and high-throughput sequencing, will revolutionize the management of clinical diagnostics for direct detection of bacteria and swift antimicrobial susceptibility testing. Importantly, such technological advancements occur in the golden age of machine learning where computers are no longer acting passively in data mining, but once trained, can also help physicians in making decisions for diagnostics and optimal treatment administration. The further potential of physically integrating new technologies in an automation chain, combined to machine-learning-based software for data analyses, is seducing and would indeed lead to a faster management in infectious diseases. However, if, from one side, technological advancement would achieve a better performance than conventional methods, on the other side, this evolution challenges clinicians in terms of data interpretation and impacts the entire hospital personnel organization and management. In this mini review, we discuss such technological achievements offering practical examples of their operability but also their limitations and potential issues that their implementation could rise in clinical microbiology laboratories.

## Introduction

Fully automated diagnostics pipeline is a seducing idea and first automated microbiology laboratories have started to be implemented world-wide (Vandenberg et al., 2018; Vandenberg et al., 2020). In parallel, machine learning (ML), a branch of artificial intelligence, has gained a foothold in many fields of clinical medicine (Topol, 2019). We actually have ML-driven tools that can make diagnosis, help clinicians in decision-making challenges (Peiffer-Smadja et al., 2020), such as the choice for a given treatment, and even empower the patients themselves to manage their healthcare (Topol, 2019). The innovative aspect of ML is that it is not a ruled-based system; ML algorithms can learn from input data and automatically make predictions or decisions.

With next-generation sequencing (NGS) techniques, we can gain information about pathogens analyzing millions of small fragments coming from their genomes and even gain insights on microbiota composition, including not-yet cultured or uncultivable organisms.

Can automation, together with new technologies, make a difference from conventional clinical microbiology tests that often require a significant amount of manual work?

What impact will such advancements have in clinical routine in terms of sample-to-result timing, taking into account that it usually takes between 24 and 48 h to obtain results in current routine laboratories (Ruppé et al., 2016)? What will such new technologies imply in terms of resources and management? Lastly, can we understand and interpret multimodal large-volume data resulting from these new technologies?

In this mini review, we will discuss these questions leveraging the benefits of technological advancements over routine diagnostics but also considering the limitations and problems by implementing them in healthcare facilities.

## Full Automation in Clinical Microbiology Laboratories

In a clinical microbiology routine laboratory, sample processing varies mostly because of the nature of the specimens (blood, urine, etc.) but also because of the diversity of pathogens that can require specific media and growth conditions. Besides pathogen identification, clinical microbiology laboratories are also in charge of providing information about the antibiotic susceptibility of pathogens to help selecting the most appropriate pharmacological regimen. Antibiotic susceptibly tests (ASTs) can be performed with different approaches (agar disk diffusion, agar gradient diffusion or broth microdilution) and can measure the minimum inhibitory concentration (MIC) of an antibiotic, that is the lowest concentration of the drug at which there is no visible growth.

To date there are only two commercially available instruments, the Copan’s WASPLab™ (WASPLab™) and the Becton Dickinson’s Kiestra TLA (Kiestra TLA), which propose automated culture-based tests including specimen streaking, slide preparation, transfer of inoculated media between instruments and automated incubators (Dauwalder et al., 2016; Bailey et al., 2019).

The WASPLab™ and Kiestra TLA are versatile technologies which can incorporate or can be combined with other diagnostic systems such as MALDI-TOF (Cherkaoui et al., 2011; Mutters et al., 2014), a key technique in modern medical microbiology to identify bacteria and fungi (Cherkaoui et al., 2010; Kaleta et al., 2011; Clark et al., 2013; Patel, 2019; Cherkaoui et al., 2020a). For example, the Kiestra TLA combined with MALDI-TOF has been shown to shorten the incubation time required to identify microbial pathogens (Mutters et al., 2014). Unlike Kiestra TLA, WASPLab™ offers an automated solution for antimicrobial disc diffusion susceptibility testing with equal or better accuracy than other available phenotypic methods (Cherkaoui et al., 2020b).

Overall, the two systems reduce the number of manual pre-analytic, analytic and post-analytic steps that are typically performed in a non-automated laboratory (Dauwalder et al., 2016). The implementation of the WASPLab™ or of the Kiestra TLA systems in clinical settings improved sample processing steps and reduced sample-to-result timing (Barake et al., 2017; Cherkaoui et al., 2019a; Cherkaoui et al., 2020c).

Since 2018, the Copan’s WASPLab™ technology has been implemented at the Geneva University Hospitals (Hôpitaux Universitaires de Genève—HUG) (Cherkaoui et al., 2020c), where it has proven offering rapid detection of vancomycin-resistant enterococci with automated incubation and digital-image based analysis system (Cherkaoui et al., 2019b) and more generally, a substantial shortening of turn-around times (Cherkaoui et al., 2019a; Cherkaoui et al., 2020a).

Full automation of diagnostic procedures can generate further advantages (Dauwalder et al., 2016; Cherkaoui et al., 2020c).

Firstly, automation increases the capability of sample processing with a better documentation and traceability. Secondly, there is a better control of the costs (e.g. reagents, medium, etc.) with reduced turn-around times thus resulting in a faster diagnosis. Thirdly, full automation permits extending the opening hours of the laboratory with a huge benefit for patient care.

Hopefully full automation will also incorporate molecular diagnostic capabilities, starting with DNA extraction, another procedure that is multi-step and requires experienced technical personnel.

Nowadays, there are plenty of DNA processing machines ranging from low to medium- and high-throughput, but not yet included in Kiestra TLA nor in WASPLab™ systems. In particular, we can distinguish two main types of instruments among commercially available ones: one that combines DNA extraction with the amplification, and the other one where extraction and amplification are performed separately (Ali et al., 2017; Shin, 2018). A technology based on automated nucleic acids (NA) analyses would be advantageous in those situations where NA-based testing is demanded on a large scale, like SARS-CoV-2 pandemic, and offering additional consolidation.

## Next-Generation Sequencing Technologies

**Figure 1:**
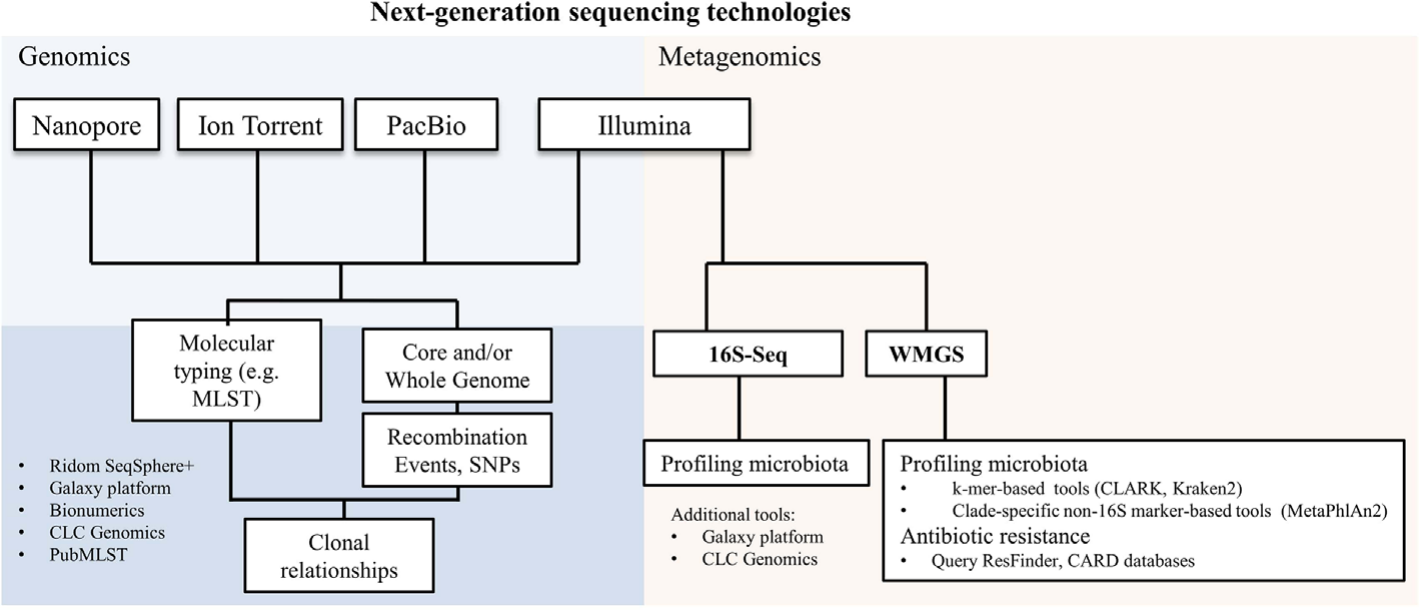
Next-generation sequencing technologies and their applications in microbiology. A non-exhaustive list of bioinformatics tools used for genomics and metagenomics analyses is reported. SNPs, single nucleotide polymorphisms; 16S-Seq, 16S-sequencing; WMGS, whole metagenome shotgun sequencing.

NGS has represented a further milestone in clinical microbiology. Today we have four main sequencing technologies, Illumina, Ion Torrent, Pacific Biosciences (PacBio) and Oxford Nanopore (**Figure 1**), which are based on a different chemistry for the sequencing and that provide different outputs in terms of number and length of the sequencing reads. Currently, Illumina short-read sequencing is the most used technology for both genomics and metagenomics, due to its sequencing depth and therefore accuracy (**Figure 1**). However, the speed of sequencing of Oxford Nanopore, combined with its ability to sequence long reads, makes it also very compelling for some diagnostic procedures (Grädel et al., 2020).

Parallel to the sequencing technological advancements, there has been an explosion of bioinformatics tools that are capable to analyze and structure the information from sequencing data.

While some of these tools, such as Galaxy platform (Giardine et al., 2005), Ridom SeqSphere+ (Ridom GmbH), CLC Genomics Workbench 20.0 (QIAGEN) and BioNumerics (Applied Maths NV - bioMérieux) display graphical user interfaces, there are many others which require coding skills for their proper and powerful usage. Most codes are publicly shared in open repositories such as GitHub and Bitbucket.

We can today apply NGS to study the core and/or whole genome (Genomics; **Figure 1**) to infer any kind of molecular typing from MLST to vaccine antigens (Pérez-Losada et al., 2018; Muzzi et al., 2019; Leo et al., 2020) and even study clonal relationships by investigating single nucleotide polymorphisms (SNPs) or genomic recombination events (Didelot and Wilson, 2015; Donner et al., 2020; Olearo et al., 2020; Pham et al., 2020; Scherrer et al., 2020).

A further important application of NGS, called metagenomics, is to profile microbiota. Metagenomics has linked microbiota species composition to a broad range of infectious diseases (Forbes et al., 2018; Egli et al., 2020), including complex nosocomial infections as ventilator-associated pneumonia (Emonet et al., 2019), suspected infectious endocarditis (Choutko et al., 2019; Kolb et al., 2019), or challenging deep-seated infections (Lazarevic et al., 2018; Foulex et al., 2019).

Metagenomics consists of two largely used experimental methods: amplicon-based (targeted metagenomics, also called metataxonomics) and whole metagenome shotgun sequencing (WMGS) (**Figure 1**). Targeted metagenomics is based on the amplification, followed by sequencing, of hypervariable regions in a target gene present in all species of the same kingdom. The gene encoding for 16S ribosomal RNA is the most used to generate taxonomic profiles. Bacterial detection by 16S-sequencing can be limited to taxonomic levels higher than the species level in some cases; besides it excludes viruses and fungi from the analyses.

Sequencing reads generated by WMGS are queried against large databases and eventually assigned to a given species not only from bacteria but also from other organisms, including Archaea, DNA viruses and eukaryotic microbes. The relative abundance of species is used to quantify a species with respect to the amount of sequencing reads.

Two main approaches are used for species identification in metagenomic sequencing datasets: k-mers- and clade-specific-marker-based. Beyond purely technical aspects, the main difference between the two methods is that k-mers-based tools, like CLARK (Ounit et al., 2015) and Kraken2 (Wood and Salzberg, 2014), can be used for large customized genome databases, while marker-based approaches, like MetaPhlAn2 (Truong et al., 2015), rely on the querying of reads against a more limited gene sequence dataset. The result is that we can detect a wider range of species with k-mers-based tools than with a marker-based approach (Leo et al., 2017). A further application of WMGS is to search for genetic antibiotic resistance by querying antibiotic resistance gene databases, like ResFinder (Zankari et al., 2012) and the Comprehensive Antibiotic Resistance Database (CARD) (McArthur et al., 2013).

Metagenomics is an appealing tool for the diagnosis of infectious diseases as it has shown to be functionally equivalent to culture techniques (Leo et al., 2017), but it can detect pathogens when they are missed by current laboratory methods (Xu et al., 2011; Mokili et al., 2013; Wan et al., 2013); it could also constitute a promising tool to be integrated in infection control and clinical epidemiology (Greninger et al., 2015).

NGS and metagenomics have not yet been automatized and the utilization of ML has been applied to different aspects, as inferring antibiotic resistance, predicting diagnosis and recurrent infection (Peiffer-Smadja et al., 2020).

## Artificial Intelligence in Automated Clinical Microbiology Diagnostics

Together with automation and NGS, artificial intelligence could also contribute to a better management of infectious diseases in helping clinicians to collect and elaborate information from clinical tests.

Computer vision that is the ability of a computer to process a digital image and identify objects represents one of the most popular examples of how artificial intelligence works. In clinical microbiology field, computer vision can be useful to improve the identification of pathogens with all those tasks that are manual and require a certain expertise like the interpretation of Gram stains (Dauwalder et al., 2016).

In fact Gram stain is an essential test which provides initial information on the presence and type of bacteria and helps in opting for a first prompt antibiotic regimen (Barenfanger et al., 2008). Smith and Kang et al. (Smith et al., 2018) realized a system where both slide imaging and Gram stain analyses interpretation were automated. They used a ML algorithm that can analyze digital images and recognize most common pathogens of bloodstream infections based on their morphologies. Their automated ML system reached an accuracy of 92.5% compared to manual classification. Similar results were obtained by adopting ML approaches to automate antimicrobial susceptibility testing and the definition of antimicrobial minimal inhibitory concentrations on the five most common Gram-negative pathogens *Escherichia coli*, *Enterobacter cloacae*, *Klebsiella pneumoniae*, *Pseudomonas aeruginosa*, and *Acinetobacter baumannii* (Smith et al., 2017).

Computer vision can ideally be applied to any type of morphologic/phenotypic test, including parasitological ones. For example, ML was applied to identify parasitic protozoa from fecal matter (Mathison et al., 2020) and malaria parasites (Florin et al., 2018).

Beyond facilitating the automation of certain tasks, ML can be of help in saving time and expenses in clinical laboratories. Burton et al. (2019) applied ML algorithms to predict whether urine samples required further testing by considering not only biological matter present in the sample (counts of white, red blood and epithelial cells) but also other factors like the pregnancy status or the age of the patient.

A recent work (Mueller et al., 2020) describes how a computer tool could analyze and validate the amplification curves generated from reverse transcription polymerase chain reaction (RT-PCR) developed for SARS-CoV-2 testing. In fact, the validation of these laboratory tests can become a laborious task for clinical personnel especially when they are performed on large scale. The consequence is to slow down the delivery of the test outcome to the patient. The algorithm developed by Mueller et al. (2020) can automatically validate SARS-CoV-2 RT-PCR tests and retain those that need particular attention.

In this perspective, such computer-based tools would help focusing on the cases that need further microbiological investigation.

## Implementing New Technologies in Real-World Settings: Considerations and Limitations

The implementation of new technologies, like automation, ML and NGS, brings several issues. Automation of a clinical microbiology laboratory is challenging until it can reach all the steps, like opening all routinely used sample containers, relying on validated incubation times and standardized antibiotic susceptibility testing (Dauwalder et al., 2016; Cherkaoui et al., 2019a; Cherkaoui et al., 2020a; Vandenberg et al., 2020).

Standardization and validation of the pre-analytical, analytical and post-analytical procedures are needed before the automated system is fully applicable to routine analyses. In this respect, tasks of the automated pipeline could be segmented and sequentially validated allowing also a better management of personnel training and implementation of instruments in the hospital routine daily life (Cherkaoui et al., 2020c). Importantly an appropriate IT system should be put in place to ensure a correct information exchange with the automated system, e.g. for the protocol of the microbiological tests/tasks to perform (Cherkaoui et al., 2020c).

Biosafety is also an important aspect that should be carefully considered when implementing a new system to appropriately handle clinical samples with biological hazard, in order to prevent accidental infections among laboratory personnel or laboratory contaminations.

ML-driven technologies are “black boxes”, meaning that the processes leading from the input to the output are unknown to the user. Therefore, although ML represents a promising tool especially in coping with large-volume complex data, the understanding of its functioning might be hard for microbiologists and clinicians who must inspect and validate the results. Furthermore, ML-driven technologies should be examined in clinical trials in order to be safely and officially incorporated in laboratory-certified operations. Thus, whether ML approaches bring an added value to diagnostics remains to be clarified, once routine implementation can be achieved and potential benefits measured.

NGS and metagenomics are neither fully standardized, nor streamlined in a way that they can smoothly integrate a routine microbiology laboratory. Some efforts to converge towards national/international validated procedures have been undertaken (Ruppé et al., 2017; Ruppé and Schrenzel, 2018; Ruppé and Schrenzel, 2019; Charretier et al., 2020). Moreover, given the large volume of sequencing data, metagenomics can demand a lot of computing resources and can be time-consuming. NGS can detect species in terms of “relative abundance” to which we should find a meaningful corresponding parameter to allow comparison with culture data.

Automated systems and NGS require the availability of suitable host facilities, trained personnel and adequate informatics infrastructure for data computation, analysis, interpretation and storage. In the absence of such factors, small hospitals are excluded from these technological advancements. Therefore, a reorganization of diagnostics laboratory networking is warranted. Although different models of automated clinical microbiology laboratories are currently implemented (Vandenberg et al., 2020), they are all characterized by a central facility with one or more satellite laboratories. While the central facility should incorporate all the current key technologies, including automatized system and NGS, satellite laboratories serve as platforms for rapid response tests (Vandenberg et al., 2020).

**Figure 2:**
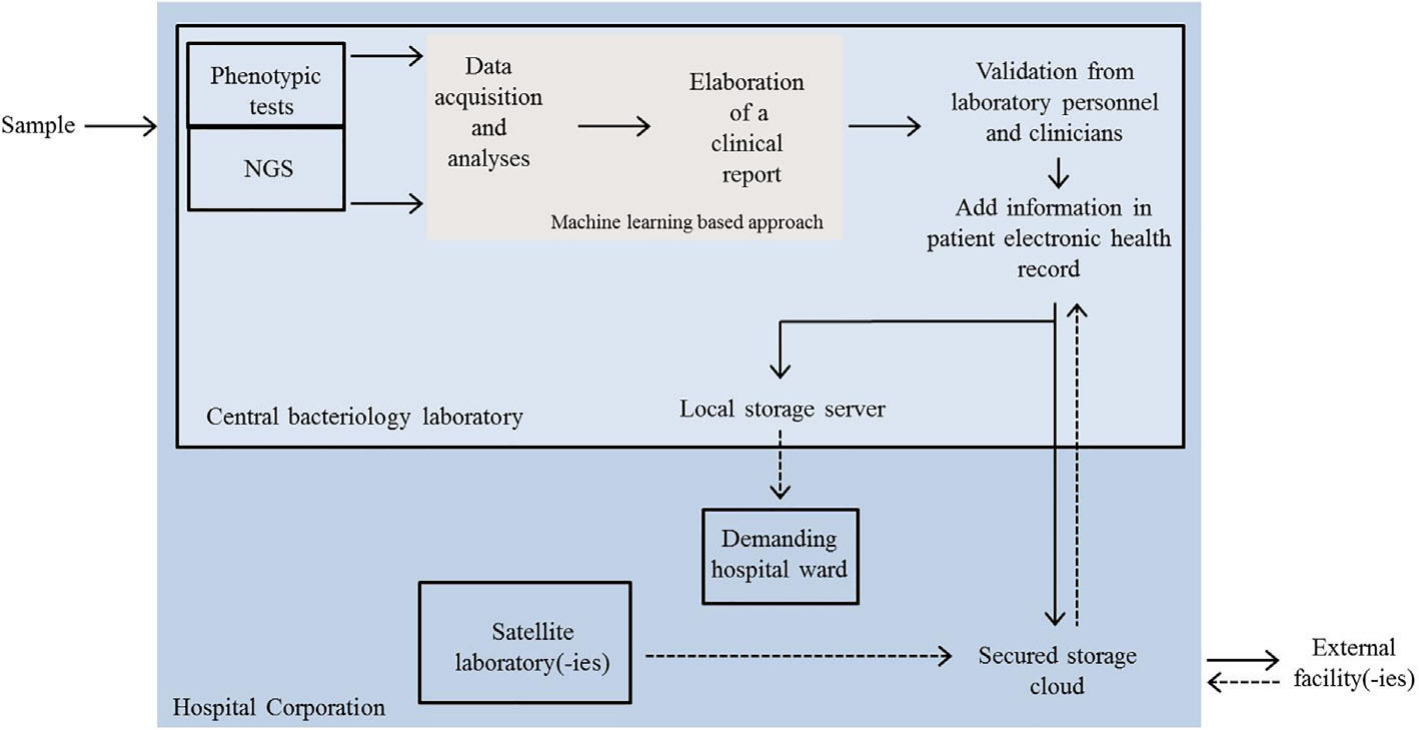
Schematic representation of a possible future scenario in the dynamics of automated clinical microbiology laboratory networking. Clinical samples are analysed by automated phenotypic tests or by NGS at the central bacteriology laboratory. Data acquisition, mining and elaboration of a first clinical report are performed by a machine learning approach. The final report is evaluated by technical and clinician experts and resulting information added to an electronic health record (EHR). EHR is then shared either internally (local server) or sent outside. Satellite laboratories and external facilities can also send the outcomes of rapid tests or other analyses to the central facility *via* a secured cloud and newly acquired information can be integrated in EHRs. NGS, next-generation sequencing.

Particular attention should be put at data communication and sharing. We can imagine that these exchanges develop at three different levels (**Figure 2**): 1) between personnel (clinicians, laboratory operators) belonging to the same hospital facility; 2) between personnel from satellite and central facilities of the same hospital corporation; and 3) between different hospitals.

For level 1), video platforms, like Zoom or Skype, provided that they respect the required medical confidentiality, might be considered for rapid clinical consultations and thus valuable instruments to keep communication during unusual situation such as the COVID-19 pandemic.

Irrespective of the type of relationships between facilities, digitalization should be accompanied with appropriate data reporting and rigorous regulation of patient data sharing.

Electronic health record (EHR) is the systematic collection of patient information in digital machine-readable format and represents a solution to data communication and interoperability between the disparate hospitals, on condition that consistent ontology definitions are used. The FAIR (Findability, Accessibility, Interoperability and Reusability) initiative principles (Wilkinson et al., 2016) should be considered to generate formal diagnostic concepts and to define standard diagnostic definitions used in EHRs. A constant curation and revision of ontologies should then be ensured especially when new technologies are introduced in routine analyses. This is the case of genomics, where information are very often not structured in a machine-readable format where new technical terms (Mascia et al., 2018) and new types of data representation are introduced. Therefore, the constitution of a data report for genomic data which is largely understood and accepted by the clinicians should be evaluated (Crisan et al., 2018).

Exchange of clinical data between infrastructures implies that patient privacy should be guaranteed at any operation level and an *ad hoc* security system should be used. Privacy-protecting technologies like homomorphic encryption and secure multiparty computations could ensure a protected environment where to store or locally analyze data, that is without the need to electronically transfer them to another informatics environment (Grishin et al., 2019). Implementation of secure computation, based on cryptographic protocol that covers the features of patients, has also been proposed for the analyses of microbiome (Wagner et al., 2016).

Initiatives like the Global Alliance for Genomics and Health (https://www.ga4gh.org/) and the European Union General Data Protection Regulation (https://eugdpr.org/), aim to harmonize legislation concerning the treatment and the protection of clinical genomic data. In Switzerland, the BioMedIT project (https://sphn.ch/network/projects/biomedit/) was established for a secure national coordination and transmission of clinical information among biomedical infrastructures.

## Conclusions

New technological advancements are going to change the appearance of clinical microbiology routine laboratories with data increasing in volume and complexity. Yet, their implementation in real clinical settings should still prove an improvement in making processes faster and cleaner than conventional workflows. Explainability and interpretability of ML-based tools are rarely addressed and independent validations should be carried out. A re-arrangement of local and regional diagnostics facilities is demanded to better cover the needs of management of automated laboratories.

## Author Contributions

SL, AC, GR, and JS conceptualized and wrote the manuscript. All authors contributed to the article and approved the submitted version.

## Conflict of Interest

The authors declare that the research was conducted in the absence of any commercial or financial relationships that could be construed as a potential conflict of interest.
